# Recent Advances in Hepatitis B Treatment

**DOI:** 10.3390/ph14050417

**Published:** 2021-05-01

**Authors:** Georgia-Myrto Prifti, Dimitrios Moianos, Erofili Giannakopoulou, Vasiliki Pardali, John E. Tavis, Grigoris Zoidis

**Affiliations:** 1 Department of Pharmacy, Division of Pharmaceutical Chemistry, School of Health Sciences, National and Kapodistrian University of Athens, Panepistimiopolis Zografou, 15771 Athens, Greece; myrtoprifti@gmail.com (G.-M.P.); moianosjim@gmail.com (D.M.); evgian@pharm.uoa.gr (E.G.); vasilikipard@pharm.uoa.gr (V.P.); 2 Molecular Microbiology and Immunology, Saint Louis University, Saint Louis, MO 63104, USA; john.tavis@health.slu.edu

**Keywords:** hepatitis B, hepatitis B virus, HBV, antiviral agents, HBV inhibitors, cccDNA, HBV life cycle, HBV treatment, nucleoside analogues, antiviral therapy

## Abstract

Hepatitis B virus infection affects over 250 million chronic carriers, causing more than 800,000 deaths annually, although a safe and effective vaccine is available. Currently used antiviral agents, pegylated interferon and nucleos(t)ide analogues, have major drawbacks and fail to completely eradicate the virus from infected cells. Thus, achieving a “functional cure” of the infection remains a real challenge. Recent findings concerning the viral replication cycle have led to development of novel therapeutic approaches including viral entry inhibitors, epigenetic control of cccDNA, immune modulators, RNA interference techniques, ribonuclease H inhibitors, and capsid assembly modulators. Promising preclinical results have been obtained, and the leading molecules under development have entered clinical evaluation. This review summarizes the key steps of the HBV life cycle, examines the currently approved anti-HBV drugs, and analyzes novel HBV treatment regimens.

## 1. Introduction

Hepatitis B is a liver disease caused by the Hepatitis B Virus (HBV). HBV belongs to the *Hepadnaviridae* family and is classified into ten genotypes (A to J) [1]. It is transmitted by exposure to infectious blood or other body fluids (e.g., semen, vaginal secretions—sexual intercourse) as well as perinatally from infected mothers to infants [2]. The acute phase of the infection can be either symptomatic or asymptomatic. Acute infections can either spontaneously resolve or proceed to chronic infections. Chronic HBV infection is among the leading causes of hepatic cirrhosis and is the single largest cause of hepatocellular carcinoma (HCC). According to the World Health Organization (WHO), over 250 million people are chronically infected, and HBV caused 887,000 deaths in 2015 [3]. The highest epidemic prevalence is present in SE Asian, African, and Western Pacific countries [4].

The hepatitis B surface antigen (HBsAg), originally known as “Australia antigen” (AusAg), was firstly identified in the serum of indigenous Australians by Baruch Samuel Blumberg in 1965 [5]. This antigen was later related with viral hepatitis [6].

The goal of the current therapeutic development is a “functional cure” defined as sustained undetectable levels of HBsAg and HBV DNA in serum, with or without seroconversion to hepatitis B surface antibodies (anti-HBs) after the end of the treatment [7]. This reduction has been associated with an improved clinical condition and significantly decreased the chance of infection rebound. Other important HBV biomarkers include serum HBV DNA, hepatitis B core antigen (HBcAg), and its antibody anti-HBc, hepatitis B e antigen (HBeAg), and anti-HBe antibody [8,9,10]. HBeAg is a secreted variant of HBcAg, and viral infections are classified either as HbeAg-positive or HbeAg-negative, with HBeAg-positive patients having higher viral titers and a more frequent and rapid disease progression [11]. These biomarkers are used to guide treatment decisions following guidelines established by the major hepatology medical societies [12,13,14].

Despite the existence of a safe and effective vaccine, no therapeutic regimen that routinely induces a “functional cure” for chronic HBV has been identified yet. This review summarizes the HBV replication cycle, the existing treatment options and their significant disadvantages, and novel therapeutic approaches that are currently the subject of extensive scientific research, with the ultimate goal of achieving a “functional cure” of the disease.

## 2. HBV Replication Cycle

### 2.1. Virion Structure and Genome

**Figure 1:**
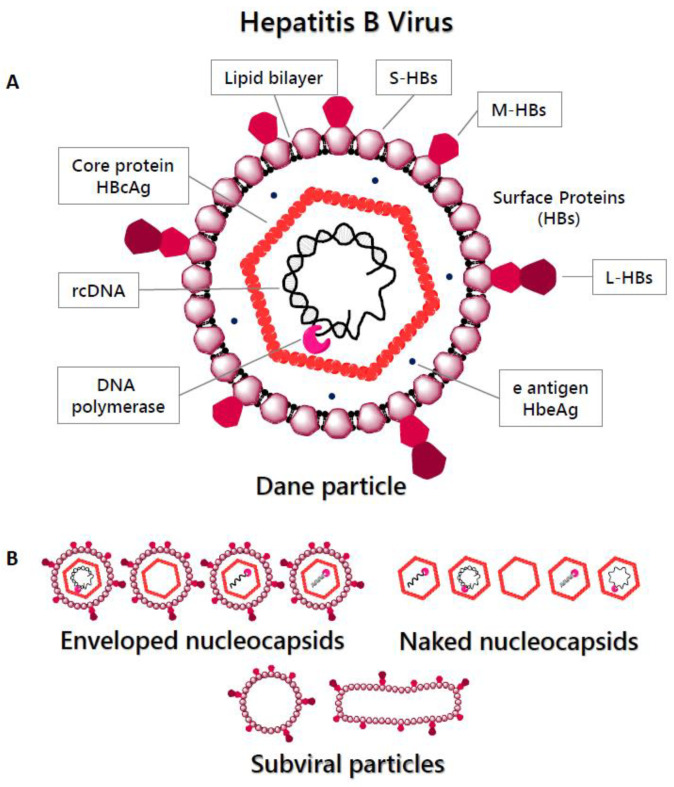
Hepatitis B Virus particles. (**A**) Infectious HBV virion (Dane particle). The lipid envelope, bearing three types of surface proteins—small (S-HBs), middle (M-HBs) and large (L-HBs)—surrounds the nucleocapsid, consisting of HBV relaxed circular DNA (rcDNA), the viral DNA polymerase (P), and the core protein (HBcAg). (**B**) Non-infectious HBV particles; enveloped nucleocapsids containing immature or defective DNA/RNA, subviral particles, and naked nucleocapsids.

HBV particles, also known as Dane particles (Figure 1A), were firstly identified by Dane and colleagues in 1970 [15]. Their shape is spherical, with a diameter of ∼42 nm. They consist of an outer envelope, which is a host-derived lipid bilayer containing three different-sized HBV surface antigens (HBsAg or HBs)—large (L-HBs), middle (M-HBs) and small (S-HBs)—surrounding the viral nucleocapsid. The nucleocapsid (∼27 nm diameter) is icosahedral and comprises the HBV core protein (HBcAg), as well as the viral DNA genome and the viral DNA polymerase (P) [16,17]. The virus also secretes a wide range of defective particles (Figure 1B), including enveloped nucleocapsids that are empty or contain defective immature genomes and subviral lipid particles containing the viral surface antigens. The subviral particles are secreted along with the infectious virions at levels that are thousands of times higher, and they play an important role in suppressing antibody responses to the virus [18]. 

**Figure 2:**
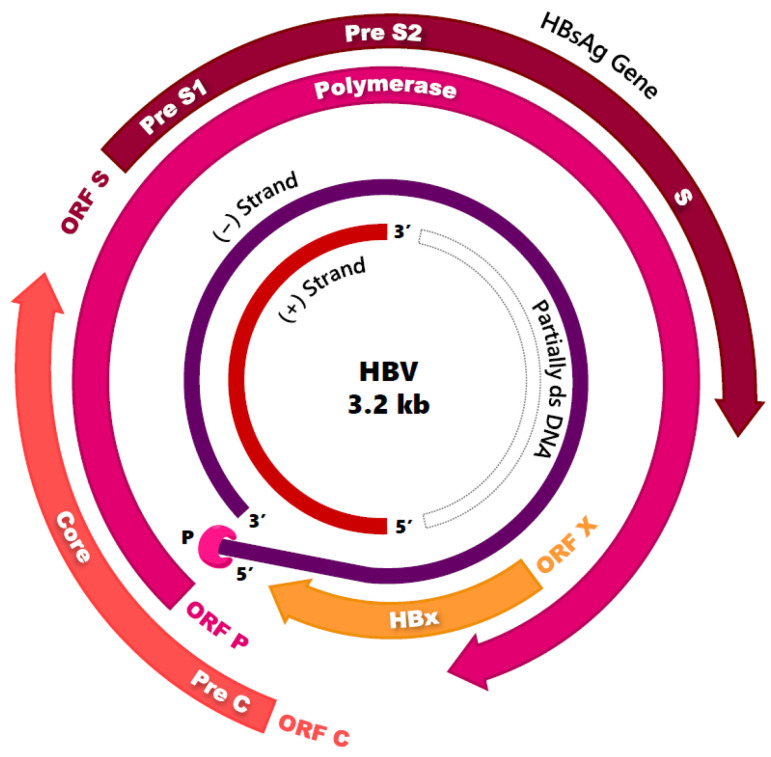
Hepatitis B Virus genome. Partially double-stranded, relaxed circular DNA (rcDNA) with four overlapping open reading frames (ORFs).

The HBV genome is a 3.2 kb circular, partially double-stranded DNA (relaxed circular DNA; rcDNA). The negative-sense, non-coding (−) DNA strand is complete and complementary to the mRNA transcripts, whereas the positive (+) DNA strand is incomplete and has a fixed 5′-end and a variable-size 3′-end [19,20,21]. The former contains four overlapping open reading frames (ORFs)—C, P, S, and X (Figure 2). These are transcribed into five RNA transcripts of varying lengths and are subsequently translated into seven functional proteins. HBcAg is produced from ORF-C, HBeAg is produced from ORF preC + C, DNA polymerase from ORF P, and HBV X protein (HBx) from ORF X. The ORF S, because of its multiple in-frame start codons, encodes the L-HBs, M-HBs, and S-HBs envelope proteins (pre-S1 + pre-S2 + S, pre-S2 + S, or S, respectively) [17,19]. This compact nature of the HBV genome results in approximately two thirds of nucleotides encoding more than one functional element [22,23]. The overlap of more than 1000 nucleotides between the P and S genes is the largest gene overlap of any known animal virus [24]. 

### 2.2. Viral Entry

**Figure 3:**
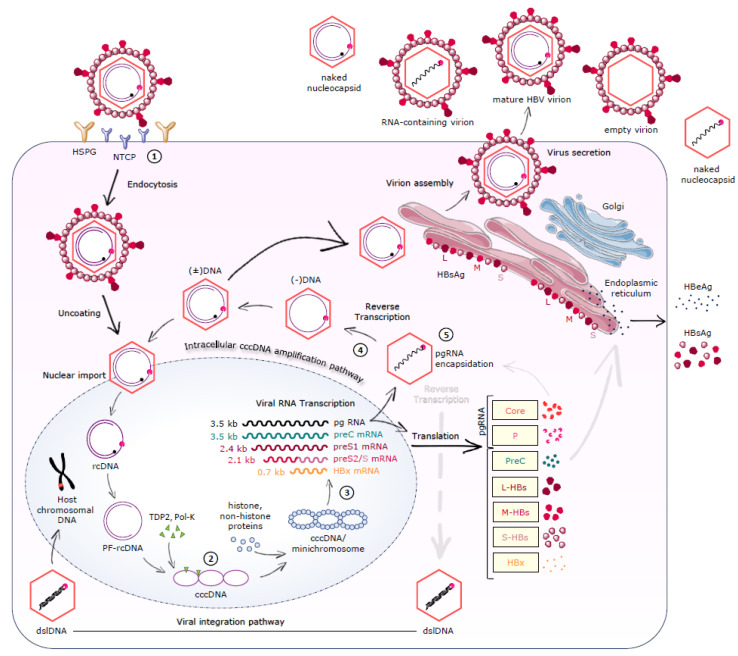
Main features of the hepatitis B virus replication cycle and potential therapeutic targets. (1) HBV entry inhibitors. Lipopeptides mimicking the pre-S1 region of HBV, monoclonal antibodies, and other small molecules under evaluation. (2) Targeting cccDNA. Damage and destruction of cccDNA via sequence-specific nucleases. Direct targeting of the HBx protein. (3) RNA interference. Small interfering RNAs (siRNAs), antisense oligonucleotides (ASOs). (4) HBV polymerase inhibitors. Reverse transcriptase inhibitors (nucleos(t)ide analogues) are part of the current treatment. RNaseH inhibitors are in preclinical evaluation. (5) Nucleocapsid assembly inhibitors or modulators can affect HBV capsid formation, reverse transcription, and pgRNA encapsidation. NTCP; Na(+) taurocholate co-transporting polypeptide, HSPG; heparan sulfate proteoglycan, rc-DNA; relaxed circular DNA, PF-rcDNA; protein-free rcDNA, cccDNA; covalently closed circular DNA, pgRNA; pregenomic RNA, preC; precore, mRNA; messenger RNA, P; polymerase, L-HBs; large hepatitis B surface protein, M-HBs; middle hepatitis B surface protein, S-HBs; small hepatitis B surface protein, HBx; hepatitis B X protein, HBsAg; hepatitis B surface antigen, HBeAg; hepatitis B e antigen, dslDNA; double-stranded linear DNA.

The HBV virion binds to the heparan sulfate proteoglycans (HSPGs) cell-surface receptors, via low-affinity and non-specific interactions. Afterwards, the Na(+)-taurocholate co-transporting polypeptide (NTCP) functions as a high affinity receptor for the recognition and attachment of the pre-S1 domain of L-HBsAg. NTCP is a liver-specific peptide that mediates the uptake of bile salts into hepatocytes, and it is also an entry receptor for Hepatitis D virus (HDV) [17,25]. Interactions between HBV and NTCP are responsible for the viral endocytosis (Figure 3). According to recent studies, a complex formed between NTCP and the epidermal growth factor receptor (EGFR) contributes to the HBV entry [26]. Due to its complicated structure, NTCP can be oligomerized, and this process seems to affect the viral internalization into the cell. Following entry, the nucleocapsid is released into the cytoplasm, followed by uncoating, and then the rcDNA is transported to the nucleus via the nuclear pore complexes [17,19,27].

### 2.3. cccDNA Formation/Maintenance

Multiple cellular factors repair the HBV rcDNA to form the episomal covalently closed circular DNA (cccDNA) that is located in the nucleus (Figure 3). Both the viral P protein which is bound to the 5′-end of the minus-polarity DNA strand [28] and the RNA primer attached to the 5′-end of the plus DNA strand are removed to leave a protein-free rcDNA (PF-rcDNA). The gaps in both strands are filled and circularized to form the cccDNA. Cellular factors believed to be involved in this process include the DNA repair enzyme tyrosyl-DNA phosphodiesterase 2 (TDP2) by presumably breaking the phosphodiesterase bond between the HBV P and rcDNA [29,30]. Another enzyme that breaks down the RNA primer at the 5′-end of the minus strand is the flap endonuclease 1 (FEN1) [31]. After removal of the proteins, the fill-in of the strands, DNA ligation, and DNA repair are conducted by other host enzymes such as DNA polymerases (κ and α), DNA ligases (LIG1 and LIG3), and topoisomerases I and II (TOP1 and TOP2) [17]. However, there is some functional redundancy among these factors, and it is not fully clear which of them function in an infected liver. The cccDNA is the template for transcription of viral RNAs. The stability of cccDNA is regulated by several cellular factors, such as the APOBEC3 protein family, that triggers cccDNA degradation [32,33]. The cccDNA is rather stable during antiviral therapy, declining by only ~1 log_10_ after more than a year of nucleos(t)ide analogue therapy [34]. However, the half-life of cccDNA has been measured at only approximately 40 days in HepG2 cells [17,35], and studies of cccDNA replacement during the reversion of nucleoside analogue resistance following the cessation of the therapy indicate that the cccDNA half-life in the liver is 16–28 weeks [36].

### 2.4. Transcription-Translation-Reverse Transcription-Nucleocapsid Assembly

Using the cccDNA as a template, the host RNA polymerase II transcribes five RNAs of different lengths: three subgenomic mRNAs of 0.7, 2.1, and 2.4 kb and two longer than genomic mRNAs of 3.5 kb (Figure 3). All of them are heterogenous, positively orientated, and have a 5′-cap and a 3′-polyadenylated tail [21]. The 3.5 kb pregenomic RNA (pgRNA) has two functions: it is the template for reverse transcription to generate the minus DNA strand and also the mRNA for the translation of the core protein and HBV P. The preC mRNA is slightly longer than the pgRNA. It contains an open reading frame that starts about 90 nucleotides upstream of the HBc ORF on the 3.5 kb pre-C mRNA and encodes the precore protein, which is converted to HBeAg upon post-translational processing in the endoplasmic reticulum (ER). The 2.4 kb mRNA encodes the L-HBs, whilst both M-HBs and S-HBs are encoded by the 2.1 kb transcript. The shortest transcript encodes the HBx protein [37,38,39]. The transcription process is regulated by four promoters (precore/core, pre-S1, pre-S2, and X) and two enhancers (Enh1 and Enh2), as well as several cis-acting negative regulatory elements [19,40,41,42,43].

HBx is the only purely regulatory protein encoded by HBV and has a multifunctional role. HBx promotes the degradation of the Smc5/6 complex (structural maintenance of chromosomes) host factor, thus enhancing the transcription of cccDNA [44,45,46,47]. Moreover, HBx represses development of the immune response to HBV infection, protecting the infected hepatocytes from immune-mediated apoptosis, and interferes with the host gene expression, facilitating the development of HCC [48,49,50].

HBV P contains four domains: the terminal protein (TP), the spacer, the reverse transcriptase (RT) domain, and the ribonuclease H (RNaseH) domain [51,52]. The pgRNA binds to P via the *ε*-stem loop located close to its 5′-end with specific motifs in the TP, spacer, RT, and RNaseH domains to form a pgRNA-P ribonucleoprotein (RNP) complex [53,54,55,56,57]. This interaction is of great importance, as it is essential for the RNA packaging into nucleocapsids and initiation of reverse transcription [58,59]. Specifically, the RNP complex is packaged within HBcAg to form immature nucleocapsids, where reverse transcription occurs, producing either rcDNA, or less often, double-stranded linear DNA (dslDNA) forms (Figure 3).

HBV replicates by reverse transcription. The RT activity of the P protein primes DNA synthesis using a tyrosine in the TP domain, covalently linking the enzyme to the product DNA. P then catalyzes the synthesis of the (−) DNA strand, which is the pattern for the (+) DNA strand synthesis, to form double-stranded rcDNA and mature DNA nucleocapsids [19,60]. During the (−) DNA strand synthesis, the RNaseH domain degrades the pgRNA template inside the capsids after it is copied into the minus-polarity DNA [61,62]. Either the newly formed mature nucleocapsids are surrounded by HBsAg and secreted non-cytolytically as virions that can infect new hepatocytes or they can re-enter the nucleus to maintain the cccDNA reservoir (Figure 3). This intracellular cccDNA recycling is likely one factor that makes the complete elimination of the HBV infection in a patient so difficult. Smaller, non-infectious subviral particles (∼22 nm in diameter) are also released from the hepatocytes in vast excess over infectious virions [17]. These include empty envelopes of HBsAg (subviral particles), virions containing RNA, or a defective DNA genome, as well as naked nucleocapsids (lacking envelope) (Figure 1) [63]. These defective particles do not participate in viral replication, although the subviral HBsAg particles help suppress antiviral immunity [18].

The dslDNA is an aberrant reverse transcription product that is able to integrate into the cellular genome early after the initial HBV infection, and it has been associated with promoting the development of HCC. The integrated HBV DNA does not replicate, but it contributes to HBsAg expression, which contributes to HBV pathogenesis and modulates the immune response [64].

## 3. Current Therapies

Two types of treatment are currently available against hepatitis B viral infection, interferon α derivatives (IFNs), and nucleos(t)ide analogues (NAs).

Interferon α (IFN-α) was first approved for the treatment of HBV infection in 1991 [65]. However, the addition of a polyethylene glycol chain to IFN-α led to significantly improved pharmacological properties. Thus, IFN-α was replaced by its pegylated counterpart, PEG-IFN-α, in 2005. There are two forms of PEG-IFN-α available today, PEG-IFN-α2a (Pegasys^©^, Roche) and PEG-IFN-α2b (Pegintron^©^, Merck). They have improved pharmacokinetics and allowed for a longer half-life, enabling a weekly administration [66]. PEG-IFN-α is administered subcutaneously and has direct antiviral as well as immunomodulatory activity [67,68,69,70]. One year of PEG-IFN-α treatment in HbeAg-positive patients led to HbeAg seroconversion in 29–32% of the patients and sustainable reduced HbsAg levels in 3–7% of the patients, 24 weeks after the end of the treatment, highlighting the effectiveness of PEG-IFN-α against HBV [2,71]. Nevertheless, the PEG-IFN-α treatment causes adverse reactions including flu-like symptoms, bone marrow suppression, fatigue, and depression, and is contraindicated for patients suffering from hepatic failure or cirrhosis [2,72]. Patient compliance is also low due to the subcutaneous administration.

**Figure 4:**
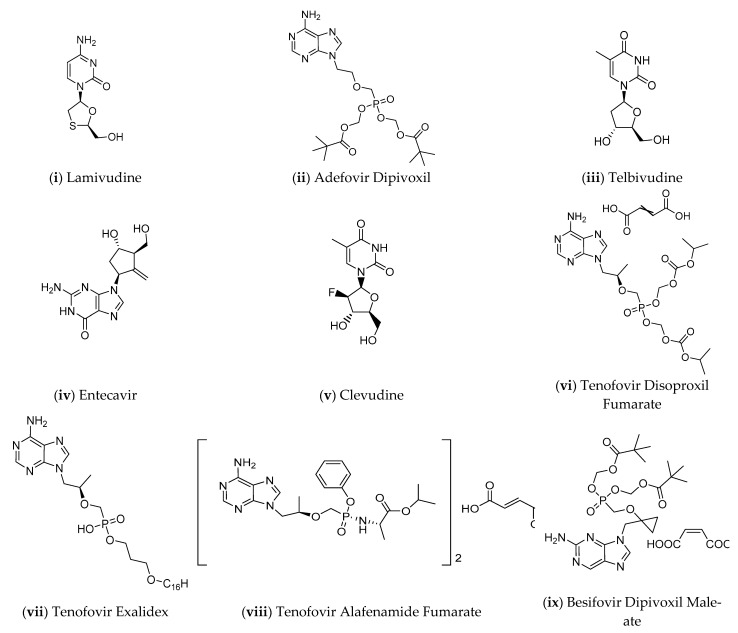
Nucleos(t)ide Analogues (NAs) approved for the treatment of hepatitis B [28,73].

Nucleoside analogues (Figure 4) inhibit the HBV reverse transcriptase activity and therefore block HBV DNA replication. The active form of most of these drugs is the triphosphate that results from their phosphorylation by hepatocyte kinases. Nucleoside triphosphate analogues are substrates for the RT. During reverse transcription, they act as immediate or delayed transcriptional terminators and prevent the synthesis of both (−) and (+) HBV DNA strands. They are administered *per os*, having acceptable pharmacokinetics and limited drug-drug interactions. NAs suppress viremia at clinically undetectable levels in up to 76% of HBeAg (+) and 93% of HBeAg (−) patients after one year of treatment. Efficacy can vary in patients with different HBV genotypes [73,74]. Although some HBeAg (−) patients can discontinue treatment with NAs, their use is essentially life-long for the large majority of patients. However, virological relapse almost always occurs. Eight NAs have been approved against the HBV, of which the current recommended ones are entecavir and the two tenofovir prodrugs, disoproxil and alafenamide [73].

The first approved NA which was effective against HBV was lamivudine (3TC, LMV, Epivir^©^, Zeffix^©^, Heptodin^©^, Hepitec^©^). It was approved in the United States of America in 1998 [73], and it is administered once daily, with few side effects. It is no longer widely used because it is less potent than newer drugs and most patients develop resistance within one to five years [65]. Data from a randomized controlled trial showed that treatment with LMV for a median duration of approximately 32 months reduced the frequency of HCC occurrence [75]. As shown in another study, receiving LMV reduced the risk of HCC even in patients with liver cirrhosis [76]. The long-term use of LMV is limited by the development of resistance associated with mutations in the YMDD (tyrosine-methionine-aspartic acid-aspartic acid) motif in the viral RT active site. A study carried out by Kwon et al. in 2013 [77] suggests that treatment in patients without mutations in this region may be continued for more than five years until the complete loss of HBsAg is achieved [77]. However, the sustained viral response obtained with LMV for more than five years showed no further decrease in the incidence of HCC [75]. In the opposite direction, Eun et al. [78] found that long-term LMV administration and subsequent prolonged viral suppression had a beneficial impact on the risk of HCC [75]. Lamivudine therapy has been confirmed to reduce liver-related mortality in patients with HBV and even in patients with co-infection with human immunodeficiency virus (HIV), especially along with other NA as combination therapy [75,79].

The next approved ΝΑ was adefovir dipivoxil (bis(POM) PMEA, ADV, Hepsera^©^ or Preveon^©^) in 2002 [73]. Given once daily, it has shown only few side effects; however, the renal function should be monitored to avoid the development of renal impairment. It is considered a second-line treatment option, except for the case of LMV resistance, where it is used as the drug of choice [65]. Although ADV monotherapy is effective in HBV patients and its long-term use reduced the rate of liver fibrosis, resistant mutations conferred decreased susceptibility to ADV [75].

Entecavir (BMS-200475-01, ETV, Baraclude^©^) was approved in 2005 [73]. It is taken once daily and causes few side effects. It is a first-line treatment with exceptional resistance profile [65], and it has been proved that it reduces the incidence of HCC [80]. The monitoring of serum alanine aminotransferase (ALT), an enzyme released by dead hepatocytes, is recommended at 6 and 12 months of treatment with ETV, since normal ALT levels are related to a reduced risk of developing HCC. Furthermore, the follow-up monitoring of serum alpha-fetoprotein as a biomarker for HCC is suggested [75].

Telbivudine (LdT, TBV, Tyzeka^©^ or Sebivo^©^) was approved in 2006 as a second-line treatment option [65]. Randomized clinical trials revealed that TBV is superior to lamivudine and adefovir in the treatment of patients with chronic HBV, regardless of the HBeAg detection [81,82,83]. In 2013, Tsai et al. [84] found that the cumulative incidence of HCC in patients who had received telbivudine was 2.5% and 4.1% at two and three years, respectively, rates similar to that of entecavir administration (3.1% and 7.5% in two and three years, respectively). TBV is associated with few side effects, including muscle toxicity and peripheral neuropathy [85,86]. Renal function should be considered when choosing between NAs, and it is worth noting that TBV can prevent nephrotoxicity [75]. At the same year, clevudine (L-FMAU, CLV, Levovir^©^ or Revovir^©^) was approved in South Korea and the Philippines. It was soon recalled due to the induction of skeletal myopathy caused by mitochondrial dysfunction [73].

Tenofovir Disoproxil Fumarate (bis(POC) PMPA Fumarate, TDF, Viread^©^) was first released in 2008. It is taken once daily and has few serious adverse effects, including dose-limiting renal toxicity. Although being a first-line treatment, TDF is also effective as a second-line rescue treatment after therapy with other nucleos(t)ide analogues has failed due to resistance evolution [65,75]. To a great extent, TDF is not susceptible to resistance development, and thus its use provides sufficient virological suppression [87]. Some studies demonstrate that patients receiving TDF have a lower incidence of HCC compared with patients receiving entecavir [75,88,89,90]. Contrarily, other studies indicate that both tenofovir and entecavir monotherapies display a comparable risk for HCC [91,92]. Tenofovir alafenamide fumarate (GS-7340-03, TAF, Vemlidy^©^) was first released in 2016, and it was developed to tackle the dose-limiting renal toxicity of TDF. The primary purpose of another analogue, tenofovir exalidex (a prodrug which is in early clinical development) is to improve the safety compared to formulations of TDF [93]. In 2017 another analogue was discovered, besifovir dipivoxil maleate (ANA-380/LB80380 maleate, BSV dipivoxil maleate, Besivo^©^), showing significantly reduced bone and kidney toxicity, compared to tenofovir [73,93].

Overall, NAs which are administered *per os,* require long-term duration of therapy, achieve better control of HBV replication, and show many fewer side effects compared to PEG-IFN-α. Treatment with PEG-IFN-α is shorter in duration and can lead to stable, off-treatment multi-log10 reductions in viral titers in about 30% of patients [2], but it is not well tolerated in many patients because of severe side effects [7,94].

## 4. Novel Therapeutic Strategies

Major scientific breakthroughs, such as the identification of the NTCP cell surface receptor, detailed knowledge gained about cccDNA formation, regulation and its epigenetic control, the mechanism of cccDNA and pgRNA degradation, and the determination of the HBx protein’s role in viral transcription, have enabled an in-depth understanding of the HBV life cycle. In addition, innovative cell and animal models have improved the in vitro and in vivo assessment of the antiviral activity and potential toxicity of novel compounds. All of the above have paved the way for investigating multiple new therapeutic targets that will lead to substantial progress toward achieving a functional HBV cure [7,95].

### 4.1. HBV Entry Inhibitors

The discovery of NTCP as the entry receptor for HBV provided key knowledge on the viral entry mechanism, thus facilitating the identification of a variety of compounds that block the viral entry into the host hepatocytes [96]. As mentioned in Section 2.2, interactions between the pre-S1 domain of L-HBsAg and NTCP are the key process for viral entry [97].

Various strategies have been proposed for the inhibition of HBV entry into hepatocytes. Small, acetylated peptides derived from the pre-S1 domain of L-HBs can effectively inhibit viral entry, exhibiting promising results both in vitro and in vivo [98,99,100]. In a recent report, novel cyclic peptides led to a significant HBsAg loss in vivo, with IC_50_ values between 0.66 and 2.54 μΜ, without affecting the physiological function of the NTCP receptor [101]. Another study revealed that peptide 4B10 was able to inhibit HBV infection in a human hepatocyte culture, with IC_50_ values in the nM range and no observed cytotoxicity [102]. The most important compound of this category is Myrcludex B (also known as Bulevirtide). Myrcludex B is a synthetic myristoylated lipopeptide consisting of 47 amino acids of the pre-S1 region. It strongly inhibits the HBV entry in the cell culture (IC_50_ = 80 pM) and in a uPA/SCID humanized mouse model of HBV infection [103,104,105]. Moreover, Myrcludex B inhibits bile salt uptake only at much higher concentrations (IC_50_ = 52.5 nM) [106]. The safety and efficacy results from clinical trials IIb are also excellent [107,108]. A liposomal formulation of Myrcludex B is under development for *per os* administration with an excellent pharmacological drug-drug interaction profile [109,110].

Several FDA-approved compounds have recently been identified as efficient inhibitors of the NTCP-L-HBs interaction. Those include the immunosuppressant cyclosporin A and its derivatives [106,111,112], the antihyperlipidemic ezetimibe [113], the angiotensin II receptor antagonist irbesartan [114], and the immunosuppressant rapamycin [115], among many drugs already in clinical use [116,117,118]. Another study identified that the green tea flavonoid epigallocatechin-3-gallate can efficiently block the NTCP-mediated viral entry [119]. Other recently identified HBV entry inhibitors include zafirlukast, vanitaracin A, proanthocyanidin, and its analogues, betulin derivatives, and novel synthetic compounds like B7 (Table 1) [117,120,121,122,123,124].

Monoclonal antibodies are also efficient HBV entry inhibitors [125,126,127,128]. Studies have proven that monoclonal antibodies not only inhibit viral entry but also block the secretion of new infectious virions from hepatocytes [129]. The combination of two monoclonal antibodies, HBV-Ab17 and HBV-Ab19, has been evaluated in phase I clinical trials, demonstrating great safety and efficacy against HBV infection [130].

### 4.2. Directly Targeting cccDNA

The ability to eliminate or inactivate HBV cccDNA has been considered as the “holy grail” of HBV treatments [93] because achieving a “functional cure” for chronic HBV infections requires the permanent inactivation or degradation of the cccDNA [131]. Steps toward disrupting cccDNA have been enabled by understanding the roles of host enzymes such as TDP2, FEN1, and Pol-K in cccDNA formation. Similarly, the identification of host cell nuclear histones and chromatin-modifying enzymes that are essential for viral minichromosome formation and function has also enabled novel drug discovery avenues. Thus, targeting the cccDNA formation has led to many novel candidates in the pipeline of HBV chemotherapy [132,133]. As referred in Section 2.3, APOBEC3 proteins can trigger cccDNA degradation. Several studies have proven that IFN-α administration induces APOBEC3 expression, resulting in the elimination of cccDNA in infected hepatocytes [134].

A potentially useful tool for the complete inactivation of cccDNA is the CRISPR-Cas9 endonuclease system to perform RNA-guided disruption and mutagenesis of cccDNA. The CRISPR-Cas9 endonuclease is complexed with a synthetic guide RNA (gRNA) that perfectly matches with the target sequence of cccDNA, resulting in the cleavage of the selected region. Therefore, to inactivate cccDNA, several gRNAs, targeting multiple different sites in the HBV genome, are required [133,135,136]. Integrated HBV DNA is also sensitive in CRISPR-Cas9-mediated inactivation, which could cause alterations in the host genome and subsequent gene malfunction [132]. Before using this genome editing approach in clinical practice, a number of serious potential issues need to be addressed. These include the incomplete cccDNA degradation, the need of a delivery system that will transfer the CRISPR-Cas9 system to all infected hepatic and extrahepatic cells, potential off-target effects, an immune response induced against the bacterial enzyme that could provoke serious toxicity, and the unpredictable effects of editing the integrated chromosomal HBV DNA [133,137].

Zinc-finger nucleases (ZFNs) and transcription activator-like effector nucleases (TALENs) are other methods that can destroy HBV cccDNA [133,138]. This treatment could slow down the growth of resistant HBV strains and increase the probability of a prolonged viral response [139,140,141], yet off-target activity, limited efficacy, effects on integrated HBV DNAs, and the potential induction of immune responses remain serious obstacles [140]. TALENs are newly developed nucleases that cleave selected DNA sequences, thus leading to gene disruptions. Several types of TALENs have been developed that target conserved regions of viral DNA among different HBV genotypes. Overall, TALENs can target and inactivate the HBV genome with a higher specificity than ZFNs and ameliorate the antiviral activity in synergy with IFN-α. Thus, a potential therapeutic strategy for the treatment of chronic hepatitis B infection is provided [140,141,142].

**Figure 5:**
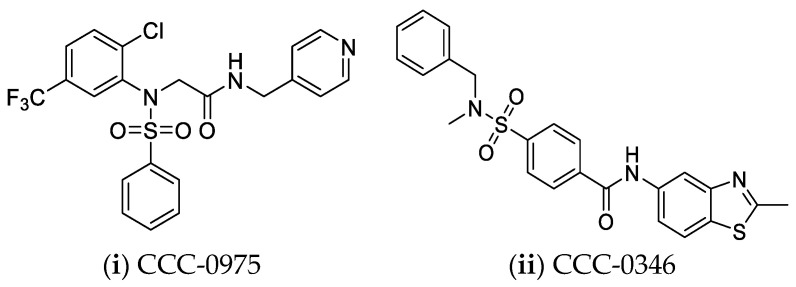
Disubstituted Sulfonamides (DSS) [144].

Another method that could contribute to the transcriptional control of cccDNA is direct targeting of the HBV X protein. HBx induces the proteasomal degradation of the Smc5/6 complex, that normally suppresses cccDNA transcription. Consequently, HBx protein inhibition will prevent the expression of all HBV transcripts from existing cccDNA molecules and suppress the formation of new cccDNA molecules [132,143]. cccDNA formation can also be directly targeted with substituted sulfonamides that interfere with the conversion of rcDNA to cccDNA (Figure 5) [144,145].

### 4.3. Immune Therapy

#### 4.3.1. Targeting Innate Immunity

**Figure 6:**
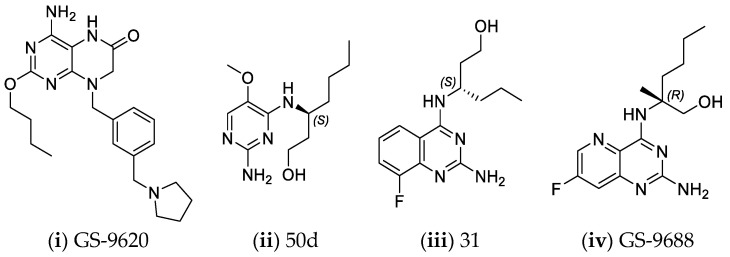
Innate immunity modulators; (**i**) Selective TLR7 agonist [163] (**ii**) TLR7/8 dual agonist [157] (**iii**) Dual TLR7/8 agonist. (R) isomer results in selective TLR8 agonist [158] (**iv**) Selective TLR8 agonist [159].

The innate immune responses constitute the first line of defense against pathogens. These systems include membrane and cytoplasmic pattern recognition receptors (PRRs). PRRs interact with specific components, essential for pathogens’ survival, called pathogen-associated molecular patterns (PAMPs), and trigger the production of pro-inflammatory factors, like cytokines from immune cells [146,147,148,149]. Thus, TLR, and RIG-I agonists can stimulate the immune response against HBV infection and contribute significantly to its “functional cure”. Several TLR7, TLR8, and TLR9 agonists are being evaluated in clinical trials [150,151,152,153]. Phase I clinical trials for TLR7 agonists RO7020531, RG7795 (ANA773), and RG7854 (Roche^©^) are currently underway. TLR7 agonist JNJ-64794964 (Janssen^©^) demonstrated an excellent safety and tolerability profile in healthy adults during a double-blinded, randomized phase I trial [154]. Phase II clinical trial results for TLR7 agonist GS-9620 (also known as vesatolimod) revealed that it is safe and well-tolerated in chronic hepatitis B patients receiving NAs, although no significant HBsAg loss was observed after 24 weeks of treatment [155]. The same compound also caused no significant HBsAg decline in combination with tenofovir in treatment-naïve patients [156]. Pyrimidine analogues were recently identified as potent dual TLR7/8 modulators (Figure 6ii) [157]. Structural modifications led to novel 2,4-diaminoquinazoline dual TLR7/8 agonists with increased potency and proved that changing the stereochemistry in one single stereocenter leads to TLR8 selectivity (Figure 6iii) [158]. TLR8 agonist GS-9688 (also known as selgantolimod) is under phase II clinical trial evaluation [150,159]. Finally, RIG-I agonist SB-9200 (also known as Inarigrivir) showed promising results in a woodchuck model of HBV infection [160,161], and phase II clinical trials demonstrated the increased benefit of combining classic antiviral treatment with immune therapy [162].

#### 4.3.2. Targeting Adaptive Immunity

The PD-1 (programmed death-1) receptor is expressed on HBV-specific T cells, and compounds that block the interactions with its physiological ligand, PD-L1, can increase the number and response of HBV-specific T cells, resulting in increased cytotoxic T cell activity against HBV-infected cells’ anti-HBV-antigen production by B cells [164,165,166]. Ex vivo studies have shown that blocking PD-1/PD-L1 interactions in chronically infected patients can partially restore the HBV-specific T and B cells’ function [167,168,169]. PD-1 antagonists have been associated with a high risk of hepatic failure [170]. On the other hand, the anti-PD-1:PD-L1 monoclonal antibody nivolumab has already been evaluated in phase I and II clinical trials in over 100 patients with advanced HCC and no hepatotoxicity incidents were observed [171,172].

### 4.4. RNA Interference—Post-Transcriptional Control

The inhibition of HBV replication by targeting mRNA production and stability is an innovative method for the therapy of chronic hepatitis B, whilst several inhibitors have made it into phase II clinical trials [7,28,93,173]. Inhibitors should bind to HBV mRNA with high specificity and therefore disrupt HBV protein expression by suppressing mRNA translation or inducing mRNA degradation. Such compounds are either small RNA interference (RNAi) molecules, antisense oligonucleotides (ASOs), or possibly even specific ribonucleic acid enzymes (riboenzymes) [133]. RNA interference is mediated by a sequence of 20–30 nucleotides, known as small interfering RNAs (siRNAs) [174]. An advantage stemming from HBV’s transcriptional profile is that multiple mRNA copies of HBV can be targeted selectively at the same time by selecting siRNAs that bind within the overlapping coding regions [133]. To date, three types of siRNAs with different modes of administration are under preclinical evaluation and/or in early-phase clinical trials [7].

An early RNAi drug against HBV, tested in human clinical trials, is ARC-520. The injection consists of two cholesterol-conjugated siRNAs, along with *N*-acetylgalactosamine (NAG) to achieve hepatocyte-specific delivery via the asialoglycoprotein receptor [39,93]. Potential use limitations are the intravenous administration, the contingent hepatotoxicity, and the off-target binding, as well as the risk of immune activation by PRRs [7]. Despite the barriers mentioned, ARC-520 seems to be very efficient in reducing HBV DNA, HBeAg, and HBsAg levels after experiments on chimpanzees [39,93]. Having passed through phase I with only few hypersensitivity reactions, it proceeded to phase II trials [28]. The co-administration of antihistamines is also recommended [28]. The next step in siRNA evolution came with JNJ-3989 (formerly ARO-HBV, phase II clinical trials), designed to target different HBV genome sites. It is administered subcutaneously, affecting transcripts from both cccDNA and integrated HBV DNA, is safe, and has not shown serious drug-drug interactions [39,93,175].

**Figure 7:**
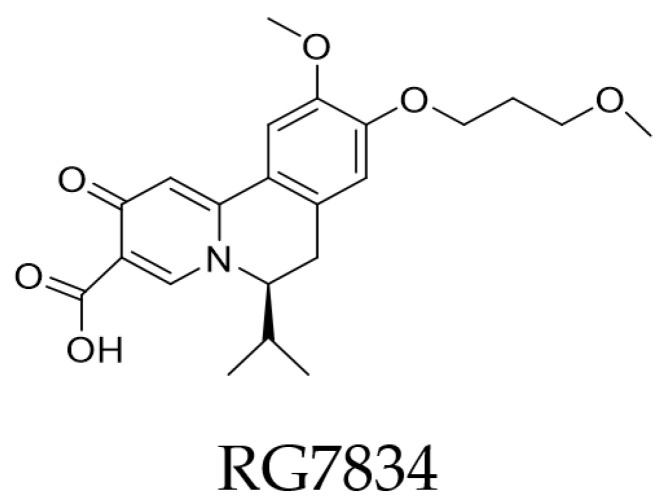
HBV transcription inhibitor [176].

The HBV inhibitor RG7834 (Figure 7), has been studied for its complicity in hepatitis B and has been confirmed not to act as an RNAi molecule, but as an HBV transcription suppressor, in a specific, unknown manner [28,176]. Furthermore, the combination of RG7834, entecavir, and PEG-IFN-α significantly decreases HBV DNA and HBsAg levels [133]. AB-729 is also an siRNA molecule which is administered subcutaneously conjugated with NAG. This compound showed an important suppression of HBsAg in mice models infected by HBV [93].

Antisense oligonucleotides are short, single-stranded fragments of nucleic acids, either DNA or RNA, that bind to the complementary sequence of viral mRNAs through base pairing. As a result, when binding to RNA, they form hybrids of DNA:RNA (antisense DNA) and duplexes of RNA:RNA (antisense RNA), respectively. The subsequent degradation of the transcribed RNA and the silencing of protein expression occur via a host RNase H-dependent mechanism [7,177,178].

### 4.5. Ribonuclease H Inhibitors

Ribonucleases H are endonuclease enzymes that catalyze cleavage of RNA sequences in DNA:RNA hybrids [179,180]. The HBV RNaseH degrades the viral pgRNA during minus-polarity DNA strand synthesis by reverse transcriptase within immature nucleocapsids [181]. Inhibiting HBV RNaseH activity results in the accumulation of long DNA:RNA hybrids and halts the reverse transcription process [62]. Consequently, newly synthesized virions are non-infectious since they contain a defective genome [182]. Thus, compounds that inhibit RNaseH are promising antiviral candidates against HBV infection.

The RNaseH catalytic site includes a ‘DEDD’ (aspartic acid-glutamic acid-aspartic acid-aspartic acid) motif that coordinates two Mg^2+^ ions. Both Mg^2+^ are essential during the RNA hydrolysis process [183,184]. All known HBV RNaseH inhibitors contain three electron donors (O or N) that chelate these two cations [185]. RNaseH inhibitors primarily belong to two chemical classes: α-hydroxytropolones (α-HTs) and *N*-hydroxyimides. The latter include *N*-hydroxyisoquinolinediones (HIDs), *N*-hydroxynapthyrydinones (HNOs), *N*-hydroxypyridinediones (HPDs), and *N*-hydroxypyrimidinediones [185,186,187,188].

One of the first identified HBV RNaseH inhibitors was *β*-thujaplicinol (compound 46, Table 2), an α-HT isolated from the heartwood of western red cedar. *β*-thujaplicinol blocks the RNaseH of HBV genotypes D and H with EC_50_ values of 5.9 and 2.3 μΜ, respectively [189]. This finding led to the design and synthesis of several novel hydroxylated tropolone analogues that suppress HBV replication in EC_50_ values as low as 0.34 μM (compound 110) [185,190,191], and with CC_50_ values up to 100 μM. Therapeutic index values were up to 200 [191]. Compound 110 has also been found to inhibit the HBV RNaseH activity, in a molecular beacon assay, and to suppress viremia in animal models [192]. Further structure-activity relationship studies on the hydroxylated tropolone ring revealed that the α-OH substitution is essential for the HBV RNaseH inhibition. Bulky substitution in positions R^1^, R^2^ and R^3^ leads to a decreased inhibitory activity, indicating that sulfonyl- or lactone substituents can increase the efficacy [190,191,193]. These findings have been validated by recent studies, which also highlighted the increased efficacy of amide-substituted α-HTs [194,195,196].

All four mentioned classes of *N*-hydroxyimides contain N or O atoms in suitable positions in order to chelate the two Mg^2+^ ions and inhibit RNaseH in the same way as the α-HTs [185]. Several analogues have been synthesized and assessed pharmacologically for their HBV RNaseH inhibitory activity, exhibiting low EC_50_ values (as low as 110 nM), limited cytotoxicity (most CC_50_ values between 25 and 100 μΜ), and TI values >300 [187,188,197,198]. One compound from this class has also been evaluated in vivo, and the results verified *N*-hydroxyimides as being effective HBV RNaseH inhibitors [192]. Finally, RNaseH inhibition is unlikely to be affected by HBV’s large genetic diversity, and RNaseH inhibitors have demonstrated great synergistic activity with antiviral compounds with a different mechanism of action, indicating that they can potentially be used in effective, combination therapeutic schemes against HBV infection [199,200]. The structures of several HBV RNaseH inhibitors identified up to date are shown in Table 2.

### 4.6. Nucleocapsid Assembly Inhibitors or Modulators

The HBV core particle is actively involved in the HBV replication cycle. It is required for the transfer of the viral genome to and from the nucleus of the infected hepatocyte, as well as for a successful reverse transcription [39]. Thus, it is a promising target for antiviral drugs [11]. New regulators or inhibitors of nucleocapsid assembly can affect various stages of the HBV replication cycle, including capsid formation, reverse transcription, and pgRNA encapsidation [28]. Based on the three-dimensional structure of capsids when they interact with a ligand, two categories of analogues have been developed [28]. 

The first category is the Class I core protein allosteric modulators (CpAMs), represented by heteroaryldihydropyrimidines (HAPs) such as GLS4, RO7049389, and Bay41-4109 [28,201]. Class I CpAMs induce the formation of deformed nucleocapsids [11,39,133,145]. The other category is that of Class II CpAMs, such as phenylpropenamides (PP) or sulfamoylbenzamides (SBA), its main representatives being: AT-130, NVR-3778, JNJ6379, JNJ0440, JNJ-632, JNJ56136379, AB-423. These compounds accelerate the assembly of morphologically normal HBV capsids that lack the viral genome [28,133,201,202]. NVR 3-778 is the first SBA derivative developed in the USA, administered *per os*, and is under phase Ia clinical trial (NCT02112799, NCT02401737), exhibiting synergistic effects in combination with PEG-IFN-α, after evaluation in HBV-infected mice with a humanized liver [7,28,39,175]. Recent in vitro studies in primary human hepatocytes have shown that JNJ-632 (SBA) and Bay41-4109 (HAP) inhibit cccDNA formation and decrease both intracellular HBV RNA and HBeAg and HBsAg levels [28]. Further in vitro studies have proved that phenylpropenamide derivatives demonstrate improved antiviral activity when combined with NAs [145]. JNJ-6379 binds to the HBV core protein and disrupts the encapsidation of pgRNA. It also blocks the cccDNA formation. This drug seems to have a very long half-life, of approximately 120–140 h [175,203,204]. ABI-H0731 marked the beginning of a new category of compounds. It is a dibenzo-thiazepine-2-carboxamide derivative, and it has been shown to cause a significant reduction in HBV DNA and RNA levels in phase I clinical trials, as a core protein modulator [39,205].

**Figure 8:**
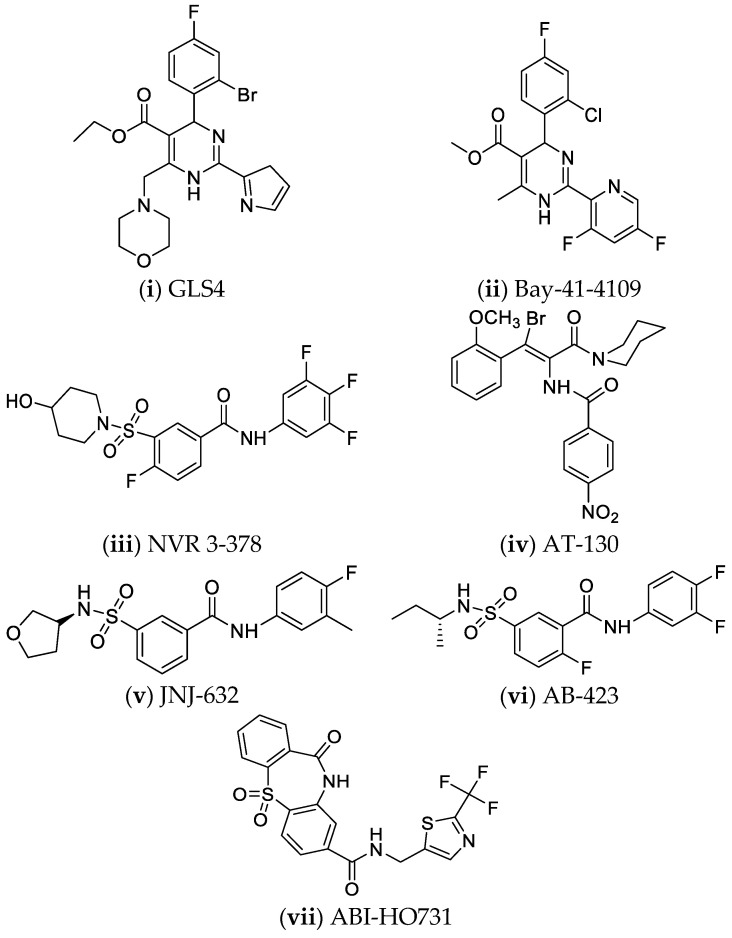
Nucleocapsid assembly modulators or inhibitors [201,205,206,207,208].

Both classes of CpAMs inhibit the release of viral particles. Thus, the amount of HBV DNA and RNA leaving the hepatocyte is reduced. They also prevent de novo cccDNA formation due to blocking the formation of functional capsids, and hence viral replication [133]. The structures of the abovementioned compounds are shown in Figure 8.

## 5. Perspectives

Hepatitis B vaccines are very effective in preventing infection, and antiviral drugs are partially effective in reducing disease progression and death from the infection. However, access to both the vaccine and the drugs remains a challenge for a large percentage of the world’s population because the majority of chronically infected patients live in developing countries, most often in sub-Saharan Africa or southeast Asia [209], with varying degrees of access to medical care. Therefore, developing an affordable and readily deliverable cure for chronic hepatitis B is urgent. It is widely believed that achieving a broadly applicable “functional cure” for chronic HBV infection will require a combination therapy using agents that target multiple different viral targets plus immune modulators that harness the power of the patients’ defenses against the virus [132,210]. Drugs to improve control and eliminate HBV will have to tackle the unique features of this infection, particularly the durability of the cccDNA during current therapies, which is the reason why the existing drugs so rarely induce a “functional cure”. Fortunately, there is a very wide range of drugs in preclinical and clinical development, so chances are high that combinations of these strategies may be found that substantially improve treatment for HBV patients.

## Figures and Tables

**Table 1:** Compounds that inhibit HBV entry in hepatocytes and their in vitro IC_50_ values for HBV infection, measured in HepG2 cell cultures.

Name	Structure	Class	IC_50_ ^1^	Ref.
Ezetimibe	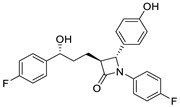	Cholesterol absorption inhibitor	18 μΜ ^2^	[113]
(−)-epigallocatechin-3-gallate	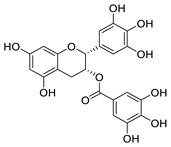	Flavonoid in green tea extract	≈10 μM	[119]
Proanthocyanidin	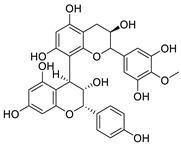	Oligomeric flavonoid, derived from extract of grape seed	7.8 μM	[122]
Oolonghomobisflavan C	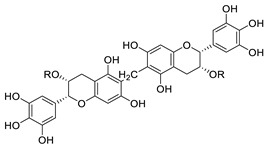	Proanthocyanidin analogue	4.3 μM	[122]
Temsirolimus	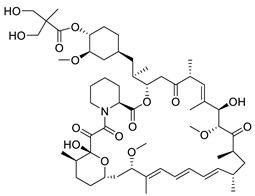	Rapamycin derivative and prodrug	7.86 μM	[115]
Compound 2	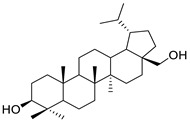	Betulin derivative	4 μM	[123]
Cyclosporin A	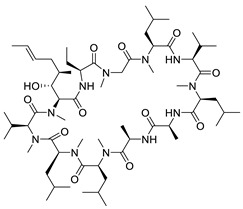	Immunosuppressant	1.17 μM	[111]
Rosiglitazone	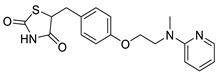	Thiazolidinedione (PPAR-γ agonist)	5.1 μM	[117]
Ciglitazone	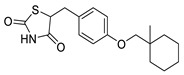	Thiazolidinedione (PPAR-γ agonist)	4.7 μM	[116]
Irbesartan	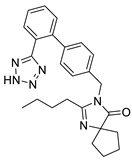	Angiotensin II receptor antagonist	3.3 μM	[114]
Zafirlukast	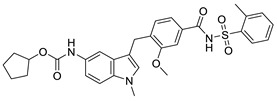	Leukotriene receptor antagonist	6.5 μM	[117]
TRIAC	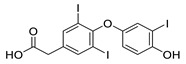	Thyroid hormone analogue	6.9 μM	[117]
B7	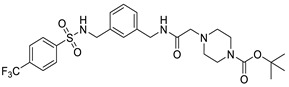	Novel synthetic compound	7.36 μM	[124]
Vanitaracin A	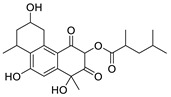	Isolated from *Talaromyces sp.*	0.61 μM	[121]

^1^ Inhibitor concentration required for 50% inhibition; ^2^ EC_50_ value (half-maximal effective concentration).

**Table 2:** HBV Ribonuclease inhibitors. The highlighted atoms chelate the two Mg^2+^ ions in the enzyme’s catalytic site. HID; *N*-hydroxyisoquinolinedione, HNO; *N*-hydroxynaphthyridinone, HPD; *N*-hydroxypyridinedione, EC_50_; half-maximal effective concentration, CC_50_; 50% cytotoxic concentration, TI; therapeutic index (TI = CC_50_/EC_50_).

**α-hydroxytropolones**					
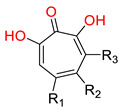 α-hydroxytropolones	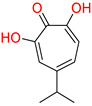 **46** [190] EC_50_ = 1.0 μM CC_50_ = 25 μM TI = 25		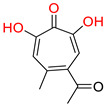 **110** [190] EC_50_ = 0.34 μM CC_50_ = 32 μM TI = 94		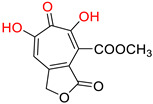 **280** [191] EC_50_ = 0.5 μM CC_50_ = >77 M TI = >154
***N*-hydroxyimides**					
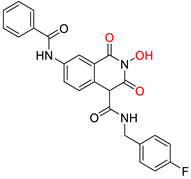 **86** [188] HID EC_50_ = 1.4 μM CC_50_ = 99 μM TI = 71		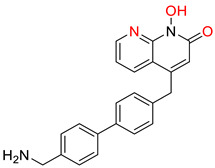 **12** [185] HNO EC_50_ = 3.4 μM CC_50_ = 7.1 μM TI = 2		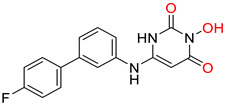 **17** [185] *N*-hydroxypyrimidinedione EC_50_ = 5.5 μM CC_50_ = >100 μM TI = >18	
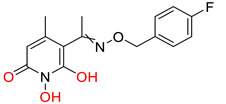 **A23** [187] HPD EC_50_ = 0.11 μM CC_50_ = 33 μM TI = 300			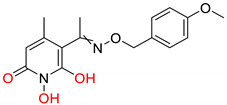 **A24** [187] HPD EC_50_ = 0.29 μM CC_50_ = 102 μM TI = 352		

## Data Availability

No new data were created or analyzed in this study. Data sharing is not applicable to this article.
